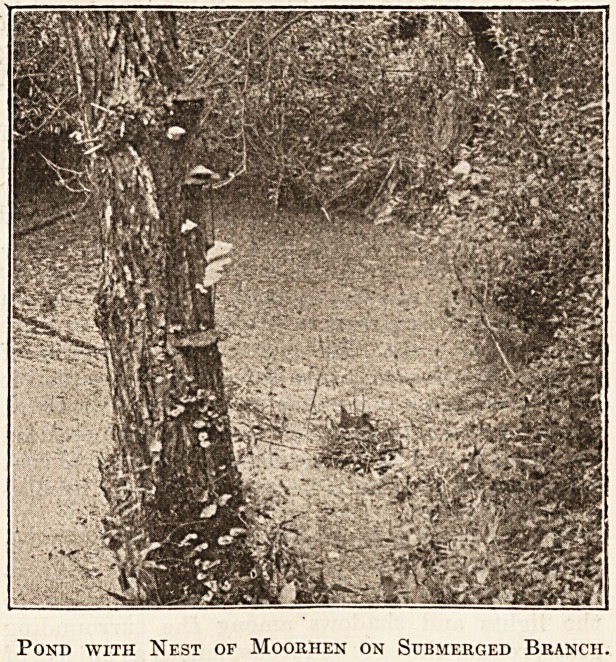# Birds and Their Nests

**Published:** 1907-05-04

**Authors:** 


### The Practitioner's Relaxations and Hobbies.

### BIRDS AND THEIR NESTS.

To lovers of natural history there is no more delightful hobby than the study of birds, notably during the nesting season. This month the poulterers' shops display the legend "Plovers' Eggs," and the town dweller can trace the advancing bird season by the gradual reduction of the prices which these luxuries at first command. The finding of the first plover's nest of the year is an event to be mentioned in the daily papers, and the rustic whose combined skill and good luck have discovered the eggs is amply rewarded. Great quantities of plovers' eggs are imported from the Continent to supplement the English market, for, alas! England has lost much of the wild marshland which is so dear to the "peewit's" heart.

Nowadays the green plover, peewit, or lapwing, as it is variously called from its appearance, voice, and curious flight, provides all the eggs of wild fowl which are eaten by epicures, though those of various sea-birds are by no means to be despised. The eggs of the black-headed gull are occasionally palmed off as those of the plover, though except in the colour of some specimens there is little resemblance between them.

Unfortunately for the peewit, its eggs possess a delicacy of flavour that makes them very much in request. As a rare dainty, they doubtless are of therapeutic value in convalescence, since anything that tempts the appetite promotes digestion all along the route.

The green plover's nest is placed in a variety of situations. Often on pasture land the bird scrapes a shallow hole and lines it scantily with dry grass. Or it may choose a ploughed field or the moorland solitudes for its home. Four, less often three, pear-shaped eggs are deposited, of an olive-green or greenish-brown colour, heavily marked with almost black patches and smaller spots. The pigment, a derivative of hæmoglobin, is partly laid down before the shell is completed, and a succeeding layer of lime salts, etc., alters its colour by obscuring it. Thus are produced the greyish underlying markings, but the final coat of paint can sometimes be partly removed by diligent scrubbing.

With all four points lying inwards, the pyriform eggs take up the least possible space. This arrangement, and their wonderful resemblance in colour to the lights and shadows among the surrounding grass, render the eggs as inconspicuous as objects of their size can well become.

Our ancestors considered the tiny eggs of the ringed plover, or ringed dotterel, and of the rarer Kentish plover indispensable to the table of the wealthy *bon vivant*. Dogs were trained to find the nests, and found so many that the Kentish plover, never very common, has as a breeding species almost vanished from England, while the ringed plover's nesting-grounds are retreating before the expansion of a host of unwholesome watering-places. Far from the madding nigger, on the shingle of some lonely beach where the bathing-machine is not, the eye is by chance arrested by four stones of exactly the same size and shape. That is all. There is often no nest whatever, and the parent takes good care to run from the eggs long before the intruder is near enough to distinguish her black and white form on the white and black pebbles. She takes to flight and becomes visible at some distance, and all you have learned is that there is a nest somewhere along that particular half-mile of beach. The photograph reproduced shows such a nest. But occasionally, when the bird has scraped a hole among the pebbles, she comes to a substratum of soil, having chosen, perchance, some spit of mud which intersects the brackish pools above the tide-mark. Here will be seen a mosaic of tiny stones

Nest of Ringed Plover.

and broken shells paving the hollow which is to hold the eggs.

It is perhaps a still harder task to find the chicks when they have hatched. A tiny fragment of black and white down, crouching motionless among the stones, gives far less indication of its whereabouts even than the eggs, which by their grouping may attract the casual glance, beautifully as they harmonise with their setting.

Another bird which nests early in the year is the moorhen, though, unlike the plovers, it continues its family affairs late into the summer. By the middle of April many pairs have begun to build. As there is then little cover but the dead stalks of last year's reed-beds, these early nests are often found as the observer follows the banks of some little stream or loiters by the margin of a secluded pond. So common is the bird that there are few suitable pieces of water in this country without their pair of moorhens, though they are not always on view. It is a shy species, and he that would see more of it than a distant form picking its way across the river, or a startled apparition splashing up under his feet out of a reed-bed, must often be content to watch in concealment for a considerable time.

Towards the end of March, when the tender passion gathers headway—the best of us, they say, make mistakes at such times—the moorhen sometimes allows nearer inspection. I have watched from a short distance half a dozen or more male birds showing off their charms and their prowess before a few hens as if human enemies were an impossible fiction. A couple of excited birds would indulge in a little exuberant horseplay—it could hardly be

Moorhen's Nest and Twelve Eggs.

called a fight—till a third rushed in and attacked one combatant, who then turned his attentions to the newcomer. Constantly changing, with never more than two pairs skirmishing at once, this tournament for the approval of the fair ones went on as long as one liked to watch it. Only occasionally would a fight begin in earnest, and, almost rearing on their tails, two stout champions would front one another in real rage. But once he was down, a few seconds of the victor's beak was enough for the beaten hero, who retired to think over what he would do next time he met his hated rival. The whole scene, with its absorbing excitement and the wild cries of all the performers, is as unlike the moorhen's usual habits as can be conceived.

For, as a rule, the bird hides in the reeds and under the bushes which fringe the stream. The nest is placed among the water plants or on a low willow overhanging the water, and is generally well hidden. Sometimes, however, as in the case figured, there is no attempt at concealment. Here an old tree, long fallen into the water, sent up an imploring arm to mark its grave, and on this open platform the bird built her nursery. Grass and ground ivy, woven into the foundations, grew green around its base, and even the lining needed frequent reminders to fade and lie flat, since everything was bathed in water. The photograph, which was taken as late as September 6, shows the bird sitting, and the track among the duck-weed along which she last swam to the nest. Perhaps the quiet garden in which the pond is placed has calmed her fears, for a pair of moorhens have inhabited it for many years in succession, and, provided the visitor does not show signs of stopping on his way past the nest, the sitting bird rarely leaves her eggs for the casual passer-by.

Such open situations as a disused boat are not unknown sites for a moorhen's nest, but the great majority are found well hidden in thick vegetation, with the surrounding reed-tops bent carefully down to hide the eggs. A clutch of twelve, as shown in such a typical nest, is not very common. Once I have met with ten, two of which were so totally distinct from the rest that no doubt two hens were laying together. Seven to nine is the usual number. The parent, on the approach of danger, slips off the nest and hides close by, and if you do light on the eggs in spite of their disguise you can be pretty sure of beating her out of the same patch of vegetation.

The moorhen is said to eat ducks' eggs. (If the duck is fool enough to let him, why, he is entitled to do so.) He does not sing, or wear fashionable clothes, or provide eider-down. His flesh is an acquired taste, and his eggs remind one of stale rain-water. He is just the most "ornery" cuss who inhabits our ponds, ditches, and rivers.

But watch, on a summer evening, his dark shape steal out of shelter, and with quaint jerky strokes step, as it were, through the water to where the

Pond with Nest of Moorhen on Submerged Branch.

reeds opposite hold his nest and patient wife. Watch his mate (you must be well hidden) paddling with her flotilla of fluffy babies up and down the water-way in search of insects and other fare meet for very Little Maries. Watch, some hard winter, a small party come up from the brook, through the meadow below the garden, and, emboldened by hunger, stand piteously on the outskirts of the crowd, hoping for a stray handful as you feed your poultry. And say whether the English landscape could spare this entirely useless bird.

## Figures and Tables

**Figure f1:**
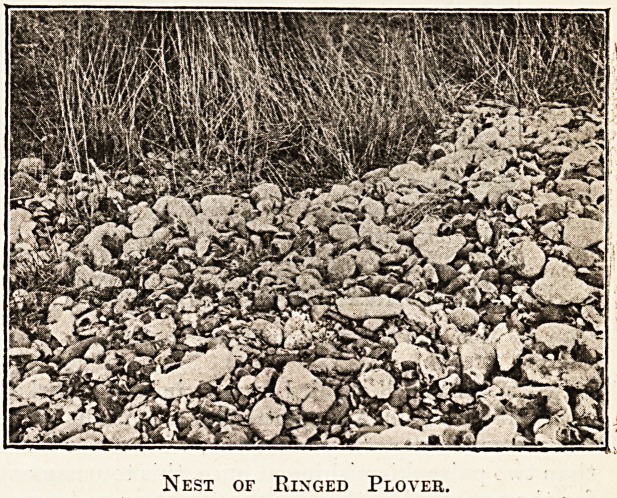


**Figure f2:**
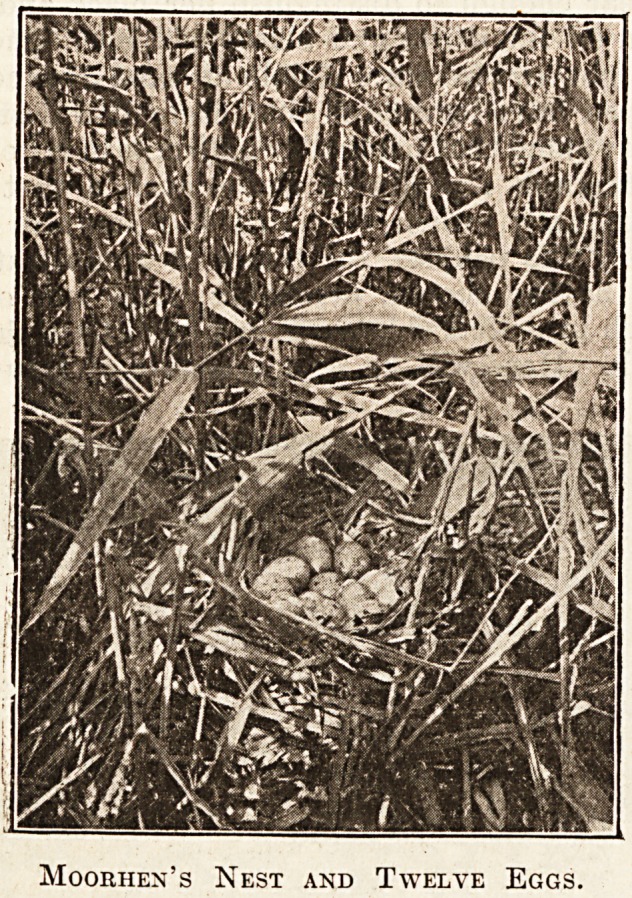


**Figure f3:**